# Role of Cyclooxygenase-2 in Head and Neck Tumorigenesis

**DOI:** 10.3390/ijms21239246

**Published:** 2020-12-03

**Authors:** Ellen Frejborg, Tuula Salo, Abdelhakim Salem

**Affiliations:** 1 Department of Oral and Maxillofacial Diseases, Clinicum, University of Helsinki, 00014 Helsinki, Finland; ellen.frejborg@helsinki.fi (E.F.); tuula.salo@helsinki.fi (T.S.); 2 Translational Immunology Research Program (TRIMM), Research Program Unit (RPU), University of Helsinki, 00014 Helsinki, Finland; 3 Cancer and Translational Medicine Research Unit, University of Oulu, 90220 Oulu, Finland; 4 Medical Research Centre, Oulu University Hospital, 90220 Oulu, Finland; 5 Department of Pathology, Helsinki University Hospital (HUS), 00029 Helsinki, Finland

**Keywords:** cyclooxygenase-2, head and neck cancers, head and neck squamous cell carcinoma, prostaglandins, inflammation, carcinogenesis, potentially premalignant lesions

## Abstract

The cyclooxygenase-2 (COX-2) is a potent enzyme that converts arachidonic acid to prostaglandins (PG), including PGE2, a key mediator of inflammation and angiogenesis. Importantly, COX-2 is activated in response to inflammatory stimuli, where it is also believed to promote the development and progression of head and neck cancers (HNC). COX-2 can mediate its protumorigenic effect through various mechanisms, such as inducing cell proliferation, inhibition of apoptosis, and suppressing the host’s immune response. Furthermore, COX-2 can induce the production of vascular endothelial growth factors, hence, promoting angiogenesis. Indeed, the ability of COX-2 inhibitors to selectively restrict the proliferation of tumor cells and mediating apoptosis provides promising therapeutic targets for cancer patients. Thus, in this comprehensive review, we summarized the reported differential expression patterns of COX-2 in different stages of head and neck carcinogenesis—from potentially premalignant lesions to invasive carcinomas. Furthermore, we examined the available meta-analysis evidence for COX-2 role in the carcinogenesis of HNC. Finally, further understanding of the biological processes of COX-2 and its role in orchestrating cell proliferation, apoptosis, and angiogenesis may give therapeutically beneficial insight to develop the management plan of HNC patients and improve their clinical outcomes.

## 1. Head and Neck Cancer: An Overview

Head and neck cancers (HNC) represent a heterogeneous group of tumors that arise anywhere in the head and neck region. Approximately 90% of these cancers develop from the squamous cell lining of the oral cavity, oropharynx, hypopharynx, larynx, or nasopharynx [1,2]. In addition, tumors can originate from other tissues, such as the salivary glands, lymphoid tissue, connective tissue, or melanocytes [3]. Epidemiologically, HNC ranks as the sixth most common cancer worldwide, accounting for about 5–10% of all cancers in Europe and North America [4,5].

The risk factors associated with HNC include, e.g., tobacco and alcohol consumption, HPV infection, poor oral hygiene, and improper diet [5]. When consumed together, tobacco and alcohol can produce a synergistic procancerous effect, whereby alcohol increases the body’s exposure to tobacco-derived carcinogens, such as nitrosamines and polycyclic hydrocarbons [4]. Thus, while smoking alone increases the risk of developing oral squamous cell carcinoma (OSCC) by ten times, both smoking and heavy drinking can increase such risk by almost a hundred times [6]. Human papillomavirus (HPV), particularly type 16 and 18, can merge with the host cell DNA and induce a malignant transformation. Interestingly, HPV is mainly associated with oropharyngeal squamous cell carcinomas (OPSCC), which are commonly diagnosed in younger patients with no clear history of smoking or heavy drinking [2,5]. Despite changes in lifestyle, HPV-driven malignancies have been on the rise over the last decade [2]. Luckily, HPV-driven cancers are more responsive to treatments, and thus, patients have a better survival rate compared to other types of HNC [4].

Unfortunately, despite the advances in cancer diagnosis and treatment, the overall survival (OS) for HNC patients has remained low. The survival outcome, however, varies depending on several crucial prognostic factors. For instance, patients with HPV-positive status show a 3-year OS rate of 82% compared with 57% in those with HPV-negative tumors [4]. Other prognostic factors include tumor site and stage at the time of diagnosis, with the most important factor being whether the patient has metastatic involvement in the lymph nodes [3]. Sadly, HNC are commonly diagnosed at later stages when the disease has already progressed and metastasized. At initial presentation, over 40% of patients have regional nodal involvement, and 10% present with distant metastases [2]. Presentation with distant metastases or a recurrent tumor spells an especially grim prognosis with a median survival of only 6–8 months [7]. In this context, recurrence represents another pressing challenge in HNC. Indeed, approximately one-third of OSCC patients relapse with locoregional recurrence. Second primary tumors are also common, with an annual rate of 4–7% [3]. The occurrence of second primary tumors could, in part, be explained by the field cancerization concept. As such, in tobacco- and/or alcohol-driven carcinogenesis, a considerable area of the mucosal tissue has been exposed to the carcinogens, and hence, may harbor mutations. Consequently, the para-cancerous, tumor-free, epithelium may already be in a premalignant change process, and could develop second primary tumors [6].

## 2. Cyclooxygenase-2An Overview

The cyclooxygenase (COX) enzyme converts arachidonic acid to prostaglandins trough two catalytic steps: First, it adds oxygen to arachidonic acid so that the unstable prostaglandin G2 (PGG2) is formed; second, it reduces PGG2 to the prostaglandin H2 (PGH2), which then can be converted, via specific synthases, to several prostanoids, such as prostaglandin E2 (PGE2), prostaglandin D2, prostacyclin or thromboxane A2 [8]. COX has two isoforms: COX-1 and COX-2. COX-1 is consistently expressed in most cells, where it mediates several physiological functions, such as platelet aggregation and production of protective mucous in the stomach lining [9]. On the other hand, COX-2 is less widely expressed, and it is mainly found in the stomach, kidney, central nervous system, and the female reproductive tract [10]. It can, however, be induced in other cell types by different stimuli, such as growth factors, cytokines, carcinogens and oncogenes, and chronic inflammation [9,11].

An increased expression level of COX-2 has been linked to carcinogenesis [11]. Elevated COX-2 levels have been found in potentially premalignant lesions and malignant tumors, including breast, lung, pancreatic, gastric, esophageal, liver, prostate, and stomach cancers. Supporting these reports, a COX-2 knocked-out mice model of familial adenomatous polyposis reduced the number of polyps, whereas mice that overexpressed COX-2 in mammary glands developed metastatic mammary cancer [11,12]. Furthermore, selectively inhibiting COX-2 in various experimental murine cancer models reduced tumor formation, growth, and metastasis [9].

**Figure 1:**
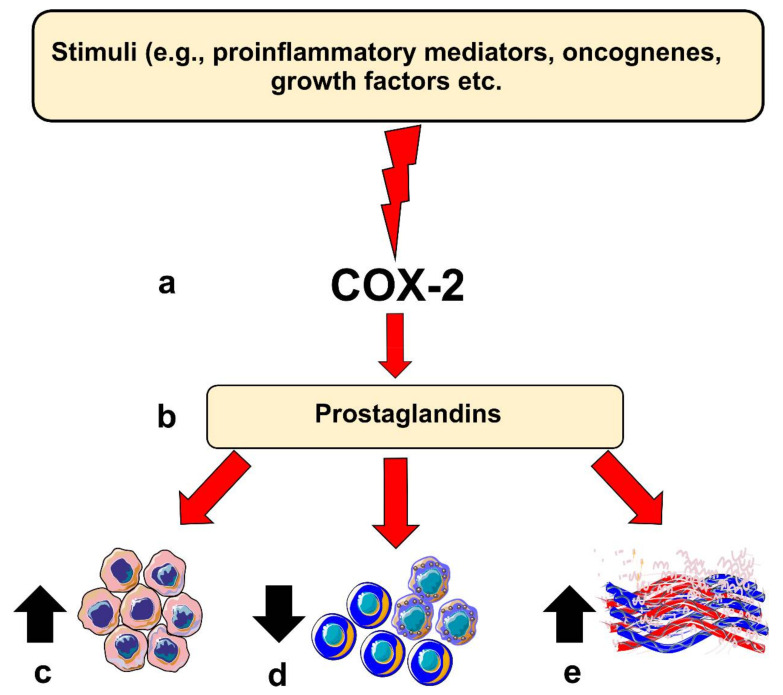
Role of Cyclooxygenase-2 (COX-2) in head and neck carcinogenesis. (**a**) Inflammatory stimuli, oncogenes or other factors can induce COX-2 expression in epithelial cells; (**b**) this results in the production of prostaglandins which can influence various protumorigenic processes, such as (**c**) enhancing anti-apoptotic response, (**d**) suppression of immune cell response, or (**e**) inducing angiogenesis in the host tissue.

COX-2 is believed to contribute to carcinogenesis in different ways. For instance, COX-2 overexpression leads to a B-cell lymphoma 2-driven anti-apoptotic effect in epithelial cells. Moreover, elevated COX-2 in these cells leads to increased production of vascular endothelial growth factors (VEGF) and the formation of networks resembling capillaries. COX-2 knocked-out mice models showed less intratumoral vascular density compared with the wild-type group [5,9]. However, COX-2 expression level per se may not directly reflect such carcinogenic potential, which could in part be mediated by its downstream pro-inflammatory products, such as PGE2 [12]. COX-2-derived PGE2 has been found to be one of the most important in carcinogenesis. Noteworthy, PGE2 levels were significantly induced in various cancer types, including OSCC [5]. PGE2 can bind to several receptors (EP1 to EP4) and acts in both autocrine and paracrine fashions, which could enhance protumorigenic processes in OSCC [5,12]. Importantly, PGE2 can suppress the immune system by inhibiting T- and B-cell proliferation and natural killer cell function; suppress the production of tumor necrosis factor-α; induce the production of interleukin-10; and stimulate regulatory T cells [8,9]. Additionally, PGE2 can also mediate chronic inflammation by promoting vasodilation and angiogenesis. Altogether, these activities, when dysregulated, may contribute to carcinogenesis (Figure 1).

## 3. COX-2 Expression in Head and Neck Cancers

The expression of COX-2 has been examined at both gene and protein levels in different types of HNC. The main findings are summarized in Table 1.

### 3.1. COX-2 Expression in Head and Neck Tumorigenesis

In general, normal oral mucosa has a very low expression of, or completely lacks, COX-2 [13,14,15,16,17,18,19,20,21,22,23,24,25,26,27,28]. However, certain tissues of the oral cavity, such as the ductal epithelial cells of salivary glands, normally express COX-2 [29,30,31,32,33]. COX-2 expression in oral mucosa is induced by exposure to tobacco and other carcinogens [25,26,27,28]. Interestingly, normal oral mucosa of smokers exhibits 4-fold more COX-2 mRNA than non-smokers, and oral cancer tissues express 50 times more than para-cancer areas [22,26]. Likewise, COX-2 is typically induced in oral potentially malignant lesions. Hay et al. found that patients with oral lichen planus (OLP) showed significantly higher levels of PGE2 compared with the control group [34].

Furthermore, patients with an erosive type of OLP had significantly higher PGE2 than the atrophic type group [34]. In agreement, Prado et al. found that the COX-2 mRNA levels were induced in oral leukoplakia compared to a normal-appearing mucosa from the same patient, as well as to healthy controls [27]. Other studies have found that COX-2 expression is gradually increased along with the transition from normal oral mucosa to cancer, where it is highest in severe dysplasia/carcinoma in situ samples [28,35].

In HNC, a large body of evidence has demonstrated the upregulation of COX-2 in malignant tumors when compared to normal oral mucosa [36,37,38,39,40,41,42,43,44,45]. For instance, one study found that COX-2 mRNA was 11-fold higher in head and neck squamous cell carcinoma (HNSCC) compared to paired normal tissue from the same patient [36]. Chan et al. found that when comparing the levels of COX-2 mRNA in HNSCC tissue, it was around 50 times higher than in the adjacent normal epithelium from the same patients and around 150 times higher when compared to normal oral mucosa from healthy controls [22]. However, some studies did not find a statistically significant difference in COX-2 levels between normal oral mucosa and tumors [38,39]. Additionally, similar amounts of COX-2 have been found in both normal oral mucosa and leukoplakia compared to OSCC samples [43]. Wenghoefer et al. found that irritation fibromas expressed less COX-2 in comparison to the healthy gingiva samples, the leukoplakia, and the OSCC samples [44]. Altogether, these reports highlight the potential involvement of COX-2 in oral carcinogenesis.

### 3.2. COX-2 Expression in Other Head and Neck Tumors

Several studies have assessed the expression of COX-2 in benign and malignant salivary gland tumors. Interestingly, Sakurai et al. found that the expression of COX-2 was group-dependent and increased from the normal salivary glands, to the salivary gland adenomas, with the highest expression detected in the salivary gland carcinoma group [31]. Furthermore, two studies found that the level of COX-2 in mucoepidermoid carcinoma (MEC) was strongly increased, whereas most of the pleomorphic adenomas and adenoid cystic carcinomas (AdCC) were COX-2-negative [46,47]. In melanomas, Nascimento et al. found that oral melanomas were consistently COX-2-positive compared with the benign oral nevi, which were completely COX-2-negative [48]. However, in another study on odontogenic tumors, both the benign and the malignant tumors expressed COX-2, however, the malignant amelocarcinoma specimens exhibited higher levels of COX-2 compared with the benign ameloblastoma samples. On the contrary, the benign ameloblastic fibromas showed higher COX-2 than the malignant ameloblastic fibrosarcomas [49]. Nevertheless, fibrous hyperplasia was found to express a very low level of COX-2 compared with other premalignant and malignant lesions of the oral cavity [50].

### 3.3. COX-2 Expression in Tumor Microenvironment

Indeed, the tumor microenvironment (TME) plays a crucial role in tumor development and metastasis [5,6]. The expression of COX-2 seems to be particularly strong at the tumor invasive-front area of the HNSCC [17,18,51]. For instance, Gallo et al. has shown that the median PGE2 protein level was 2.36 μg/mg in the tumoral core compared with 3.85 μg/mg in the invasive-front area of HNSCC [52]. In this study, the cohort included 52 surgical specimens from laryngeal, oral cavity, and oropharyngeal SCC. Positive expression of COX-2 has also been found in the tumoral surrounding stroma of HNSCC, most notably in the inflammatory cells, fibroblasts, and endothelial cells [17,19,41,49,51,53,54,55,56]. Höing et al. compared the expression of various markers, including COX-2, between stroma and tumor nests in 110 laryngeal squamous cell carcinoma (LSCC) patients [57]. Interestingly, and in contrast to the other markers, COX-2 was expressed more in the tumor nest (53%) than in the stroma (39%) of the LSCC patients. Furthermore, this study revealed that tumoral, but not stromal, COX-2 expression correlated with lymph node metastasis and reduced patients’ survival. Hence, since COX-2 can influence immune cell recruitment, the authors proposed that COX-2 could play an important role in establishing tumor-stromal cell crosstalk [57].

## 4. COX-2 Expression and Cancer Staging

The TNM Classification is a system used for classifying solid tumors and can be employed to assist in prognostic cancer staging [58]. The T stands for tumor size; N stands for nodes, and it describes the regional lymph node involvement of the tumor; and M stands for metastasis, and it informs whether the tumor has metastasized to distant tissues. Cancer stages are usually divided into stages (0 to IV), with stage 0 having the score Tis (i.e., carcinoma in situ), with the numbers increasing gradually (T1-T4, N1-N3, and M1) with the most advanced stage being IV [58].

Importantly, many prognostic studies indicated a significant relationship between the level of COX-2 and the T-stage in patients with HNC. Among these, three studies concluded that induced immunoexpression of COX-2 was significantly associated with the T-stage in OSCC patients [41,59,60]. Similarly, Loong et al. found that advanced T-stage tumors of patients with nasopharyngeal carcinomas (NPC) showed stronger COX-2 expression compared with the lower T-stage tumors [61]. In LSCC patients, COX-2 expression was, likewise, more prevalent in the T3 and T4 tumors than in the lower T1 and T2 tumors [62,63]. Furthermore, Yang et al. reported a similar observation that COX-2 expression was significantly correlated with advanced T-stage in hypopharyngeal SCC (HPSCC) patients [64]. Xu et al. [65] found a significant relationship between COX-2 expression and T stage when looking at NPC samples. However, a correlation between COX-2 immunoexpression and T-stage was not found to be statistically significant in some studies about OSCC [25,51,55,66,67] LSCC [16,68,69], HPSCC [24], MEC [70], NPC [71,72,73], HNSCC [74,75], and tongue squamous cell carcinoma (TSCC) [76]. Nonetheless, some of these studies found a significant correlation with at least one other prognostic parameter, such as the N-stage [24,51,70,71].

Specifically, the N-stage was significantly correlated with COX-2 immunoexpression in OSCC [45,51,59,77,78], LSCC [62], HPSCC [24,64], MEC [70], NPC [65,71,79], TSCC [37], OPSCC [80], and HNSCC [52,81]. Like with T-stage studies, some studies revealed a statistically non-significant relationship between the N-stage and COX-2 expression. These studies included samples from patients with OSCC [20,41,55,60,66,67,82,83], LSCC [16,68], NPC [61,72,73,84], HNSCC [28,74,75,85], and TSCC [76,86]. Almost all the included studies have not assessed the M-stage separately, instead, it was included in the cancer stage. In this regard, a possible link between tumor stage (I-IV) and COX-2 expression has been evaluated in different HNC. On one hand, COX-2 immunoexpression was significantly correlated with the cancer stage in OSCC [20,23,59,60,87], LSCC [62,63], MEC [70], HNSCC [52,81], and TSCC [40,88]. On the other hand, such a link between cancer stage and COX-2 immunoexpression was not statistically significant in some studies that examined patient samples from OSCC [39,41,51,55,66,67,78], TSCC [37,76], LSCC [69], HNSCC [74,75], NPC [52,70,71,82], and glottic cancer [89].

In a meta-analysis study conducted by Yang et al., COX-2 immunoexpression levels were significantly associated with N-stage and cancer stage, but not with T-stage. However, the subgroup analysis revealed that such a significant correlation between N-stage and COX-2 was only seen in patients with OSCC, but not in other HNSCC [90]. For the cancer stage, the correlation was significant in OSCC patients, as well as in no site-specific HNC patients, but not in patients with LSCC or NPC [90].

## 5. COX-2 Expression and Cancer Grading

Cancer grading is a delineation of the microscopic features of the tumoral cells and tissue. Low-grade, well-differentiated tumors exhibit histological structures that relatively well mimic the normal tissue. On the contrary, higher-grade tumors (i.e., poorly differentiated or undifferentiated tumors) have more abnormal appearing structures, and they tend to be more aggressive and have a worse prognosis [58]. Unlike TNM-staging, most studies did not find any significant correlations between cancer grade and immunoexpression of COX-2, including studies on OSCC [25,39,51,54,55,60,67,78,91], HPSCC [24,64], LSCC [16], MEC [70], TSCC [37,88], HNSCC [52,74,81,85], NPC [72,73], and glottic cancer [89]. Interestingly, the significant correlation was only seen in a few studies, including OSCC [23,41,45] and LSCC [62,63].

## 6. COX-2 Expression and Survival Outcomes

### 6.1. COX-2 Expression and Overall Survival

Itoh et al. found that OSCC patients with COX-2 overexpression had worse OS in the univariate analysis, however, COX-2 was not an independent prognostic factor in the multivariate analysis [51]. In a univariate analysis of an LSCC cohort, patients with elevated cytoplasmic expression of COX-2 had shorter OS [63]. Pan et al. showed that Cox-2 was overexpressed in 75.7% of NPCs, and this was associated with the worse OS on both univariate and multivariate analyses [92]. In the same manner, several other studies found a significant association between higher COX-2 level and reduced OS both in univariate and multivariate analyses, including studies on OPSCC [93], OSCC [94], LSCC [62], HNSCC [28,52], NPC [65,84], HPSCC [64], and glottic cancer [89]. Interestingly, Kyzas et al. found that co-expression of COX-2 and VEGF-C meant a significantly shorter OS, which was also an independent prognostic factor in the multivariate analysis [81]. Furthermore, Gallo et al. showed that HNSCC patients with higher PGE2 tumor levels had significantly shorter OS estimates in Kaplan–Meier analysis [52].

On the contrary, Ranelletti et al. reported that LSCC patients with COX-2 positive tumors had a longer OS compared to patients with COX-2 negative tumors. In this study, the 5-year OS rate for patients with COX-2-positive tumors was 100%, whereas it was 34% for those with COX-2-negative tumors [69]. In the multivariate analysis, COX-2 retained its significance as an independent prognostic marker. The authors concluded that COX-2 is overexpressed in less aggressive, low-grade laryngeal SCCs, whereas its expression is lost as the tumors progress to a more malignant phenotype [69]. Other studies found no relationship between COX-2 expression and OS, including studies on NPC [71,72,73], TSCC [67,76], OSCC [39,60,66,82,95,96], HNSCC [85], OPSCC [80], and AdCC [32].

### 6.2. COX-2 Expression and Disease-Specific Survival

In two OSCC studies, patients with higher COX-2 expression had a significantly shorter 5-year disease-specific survival (DSS) [59,60]. In contrast, Loong et al. found that DSS was shorter in patients with low COX-2 expression compared to patients with moderate or strong expression scores [61]. However, this study had a small sample size, and hence, a multivariate analysis could not be performed. Four other studies found no correlation between COX-2 expression and DSS, including patients with LSCC [68], TSCC [97], tonsils, and base of tongue SCC [98].

### 6.3. COX-2 Expression and Disease-Free Survival

COX-2 expression was found to correlate with disease-free survival (DFS) in HNSCC patients. For instance, Chen et al. found a higher recurrence rate in LSCC patients expressing high COX-2 levels compared with those with low COX-2 expression [62]. In a univariate analysis, Pan et al. found that NPC patients exhibited a significant correlation between COX-2 expression and DFS [92]. In the multivariate analysis, multiple variables, including COX-2, were combined into a principal component (Z), which was an independent prognostic factor in NPC. However, COX-2 expression was not assessed separately [92]. In HNSCC patients, the 5-year relapse-free survival rate in the univariate analysis was worse in patients who had elevated expression of COX-2, however, this was not statistically significant in the multivariate analysis [75,80]. Pannone et al. examined a cohort of OSCC patients and found that COX-2 overexpression was correlated with reduced DFS in the univariate analysis, however, multivariate analysis was not performed [39]. Similarly, Kourelis et al. reported a lower recurrence rate in LSCC patients with higher levels of COX-2 immunostaining, although the multivariate analysis was not performed [68]. Interestingly, higher COX-2 levels were associated with a poor outcome in chemotherapy-naïve OSCC patients compared to those who had received chemotherapy [99]. In the multivariate analysis, Itoh et al. reported that COX-2 overexpression was an independent prognostic factor for shorter DFS in OSCC patients [51]. In agreement with this study, Gallo et al. delineated that HNSCC patients with low or absent COX-2 expression had better DFS than patients with overexpressed COX-2 status, which was also true in the multivariate analysis [52]. However, and despite the aforementioned evidence, several studies found no correlation between COX-2 and DFS, including studies on NPC [71,72,73,84], OSCC [41,45], HNSCC [81,85], TSCC [67,88], glottic cancer [89], and LSCC [100].

### 6.4. Meta-Analyses of COX-2 Expression and Survival

Two meta-analysis studies examined the prognostic value of COX-2 expression. Wang et al. performed a meta-analysis of 12 studies encompassing 979 OSCC patients. They found that patients with positive COX-2 status had a poor OS rate (hazard ratio (HR) = 2.23) compared with the COX-2-negative group [101]. An analysis conducted by Yang et al. included 29 studies with a total of 2430 patients with HNSCC [91]. They found that positive COX-2 expression was associated with poor outcomes in OS, relapse-free survival, and DFS (HR = 1.93; 2.02; 5.14, respectively). When the authors conducted a subgroup meta-analysis, COX-2 expression predicted reduced, statistically non-significant, survival time [91].

## 7. COX-2 Polymorphisms and Risk of Cancers

**Figure 2:**
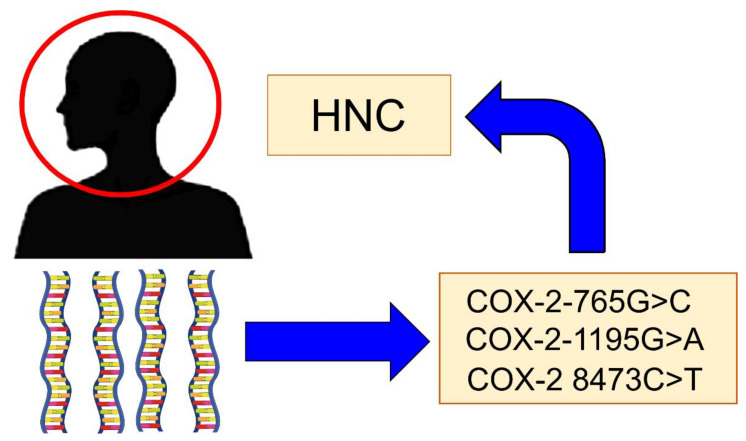
The main Cyclooxygenase-2 (COX-2) polymorphisms are implicated in the risk of head and neck cancer (HNC).

There are some genetic polymorphisms of COX-2 that have been implicated in the risk of developing HNC (Figure 2). The main polymorphisms are:

### 7.1. COX-2-765G>C

The COX-2-765G>C is a functional polymorphism that disrupts the binding site of stimulatory protein 1 (Sp1), but creates a binding site for E2 promoter binding factor 1 (E2F1), leading to stimulated transcription activity, which could enhance the cancer risk [102,103]. Lin et al. found that the GC and CC genotype was protective against OSCC when compared to the GG genotype in a study that included 297 OSCC patients and 280 healthy controls [102]. In another study on OSCC, Mittal et al. analyzed a single locus and found no significant difference between 193 OSCC patients and 137 controls in the 765 G>C allele frequency [104]. However, in the multivariate logistic regression analysis, the −765 G>C genotype appeared to be protective with an odds ratio of 0.71. Thus, they concluded that the −765 G>C and CC variant might be protective against OSCC compared to the GG variant [104]. However, this was in contrast to another study, in which the GG genotype was more frequent in controls than in OSCC patients (94.66% vs. 73.3%), and thus, could be protective against OSCC. Moreover, the study found that both the GC and the CC genotypes were associated with a significantly increased risk of OSCC [105]. Nonetheless, another two studies found no evidence for the role of −765G>C polymorphisms in the risk of developing OSCC [106,107].

### 7.2. COX-2-1195G>A

The COX-2-1195G>A polymorphism has also been suggested to influence the risk of oral cancer. The −1195A allele displays an increased transcriptional activity of the COX-2 gene compared to the −1195G allele [108]. Mittal et al. found that −1195GA genotype was relatively higher in OSCC patients compared to the controls, which seemed to confer an increased risk of tobacco-related oral carcinogenesis [104]. Chiang et al. found that the AA genotype was significantly associated with OSCC when compared to the GG genotype and had a 1.55-fold increased risk of OSCC [107]. However, two studies found no association between different COX-2-1195G>A polymorphisms and head and neck cancer risk [106,109].

### 7.3. COX-2 8473C>T

The 8473 C>T polymorphism is located in the 3′ UTR region of the COX-2 gene, and the T to C change may affect the stability and the secondary structure of the mRNA of COX-2 [110]. COX-2 8473 C>T polymorphisms have also been assessed in patients with HNSCC. Although there was no significant difference between healthy controls and OSCC patients in the single-locus analysis, the CT genotype was less frequent in patients than controls [104]. Campa et al. investigated the SNPs, including the 8473 C>T polymorphism in 811 patients with upper aerodigestive tract cancers, including OSCC, LSCC, and OPSCC [110]. The authors indicated a possible association between esophageal cancer and the 8473C>T polymorphism.

### 7.4. Meta-Analyses of COX-2 Gene Polymorphisms and Risk of Cancer

Three meta-analyses assessed the potential association between COX-2 gene polymorphisms and the risk of HNC. Deng et al. reported a significantly increased risk of HNSCC in three genetic models of COX-2 polymorphisms. However, the odd ratios were small, and not all models showed an association with HNSCC, which could result from sample size that was too small [111]. Li et al. investigated the polymorphisms: +837T>C, −765G>C, and −1195A>G among seven clinical studies, including a total of 2296 oral cancer patients. Interestingly, the authors found that the +837T>C and the −765G>C polymorphisms are related to the susceptibility of oral cancer and that the gene frequencies in the case group compared to the control group were significantly different both in the allele model and the dominant model [112]. On the other hand, a meta-analysis by Leng et al. included eight case control studies and found no association with either the 8473T>C or the −765G>C polymorphism in the risk of HNSCC. However, they found an association between the −1195G>A polymorphism and HNSCC risk in the pooled result from the crude data in certain models (AA vs. GG, AA vs. GA, and AA vs. GG + GA) [113].

## 8. COX-2 and Cancer Biomarkers

Several studies investigated the potential correlation between COX-2 and other biomarkers in HNC. However, no significant correlation was found between COX-2 and p53 [19,67,91], Ki67 [19,54], CD68 [54], epidermal growth factor receptor [72,73,84], E-cadherin [37], C-erbB2 [84], p-ERK1/2 [25] or mast cell density [95]. Nevertheless, a positive correlation has been found in a limited number of studies between COX-2 and HGF [33], EP300 [62], matrix metalloproteinase 2 [63], prostate-specific membrane antigen [94], DNA topoisomerase II α [18,77], NF-κB [41], H-Ras [23], cytoplasmic, but not nuclear HuR expression [16,47,60], CD4 + CD25 + Foxp3+ regulatory T cells [75], tumor-associated tissue eosinophilia [87] or platelet-lymphocyte ratio [66].

Importantly, a significant correlation between COX-2 and VEGF was reported in HNSCC [52,59,81,95]. The VEGF family plays a crucial role in tumor-associated angiogenesis and lymphangiogenesis. Cancer cells can secrete VEGF-C and VEGF-D to induce intratumoral and peritumoral lymphangiogenesis, as well as tumor neovascularization [52,59]. COX-2 is believed to stimulate VEGF expression (e.g., VEGF-A and C), and hence, both are associated with lymph node metastasis and tumor angiogenesis [52,59,81]. Co-expression of both factors may also negatively impact the survival of HNSCC patients [76,81].

## 9. Conclusions

To summarize, there is enormously growing evidence supporting the involvement of COX-2 in tumor-initiating and tumor-promoting events for several solid tumors, including HNC. Furthermore, elevated COX-2 levels were also documented in potentially premalignant lesions of the oral cavity. It is also acknowledged that COX-2 plays vital role in regulating tumorigenesis-related processes, such as apoptosis, angiogenesis, and immunomodulation [5,8,9]. Therefore, there is considerable potential for COX2-based therapeutics, such as COX-2 inhibitors, to serve as either adjuvant therapeutics increasing the overall response rate, or as targeted therapeutics for HNC patients. Recently, Janakiraman et al. determined that inhibition of COX-2 via celecoxib promoted apoptosis in paclitaxel-resistant oral cancer cells, both in vitro and in vivo. Thus, the authors recommended the use of the COX-2 inhibitor celecoxib, in combination with paclitaxel, for the management of paclitaxel-resistant oral cancer cells [114]. However, as aforementioned in this review, some studies showed no significant association between COX-2 and HNC, which, therefore, necessitates more studies with larger sample sizes across different populations. In conclusion, further in vitro and in vivo model studies of COX-2 role in cancer, paralleled with clinical trials, could provide promising therapeutic targets in HNC, and improve the patients’ clinical outcome.

## Figures and Tables

**Table 1:** Summary of COX-2 expression in different types of head and neck cancers.

Cancer Type	Expression	Main Findings	References
HNSCC	Gene expression	COX-2 mRNA was 11-fold higher than normal control	[36]
HNSCC	Gene expression, Immunoexpression	COX-2 mRNA was 50 times higher than para-cancer tissue; and 150 times higher than in healthy controls	[22]
HNSCC	Gene expression, Immunoexpression	No statistically significant difference in COX-2 levels between cancer and control	[38,39]
OSCC	Immunoexpression	Similar levels of COX-2 were found in both normal oral mucosa and leukoplakia	[43]
OSCC	Gene expression	Irritation fibroma had less COX-2 than cancer tissues	[44]
Salivary gland carcinomas	Immunoexpression	COX-2 had the highest expression in the salivary gland cancers, including MEC, AdCC, and pleomorphic adenomas	[31,46,47]
Oral melanoma	Immunoexpression	Tumors were COX-2-positive compared with the benign oral nevi, which were completely COX-2-negative.	[48]
Odontogenic tumors	Immunoexpression	Amelocarcinoma patients had higher levels of COX-2 compared with the benign ameloblastoma group. Malignant ameloblastic fibrosarcomas had less COX-2 than benign ameloblastic fibromas	[49]
OSCC	Immunoexpression	COX-2 was increased in the OSCC group compared with the hyperplastic group	[50]
HNSCC	Immunoexpression	The PGE2 protein level was induced in the invasive-front area more than in the intratumoral core	[52]
HNSCC	Immunoexpression	Positive expression of COX-2 was found in the para-cancer stroma, mostly in the inflammatory and endothelial cells	[17,19,41,49,51,53,54,55,56]
LSCC	Immunoexpression	COX-2 was more induced in the tumor nest (53%) than in the stroma (39%). Furthermore, tumoral COX-2 expression correlated with shorter survival outcome	[57]

AdCC, adenoid cystic carcinomas; COX-2, Cyclooxygenase-2; HNSCC, head and neck squamous cell carcinoma; OSCC, oral squamous cell carcinoma; LSCC, laryngeal squamous cell carcinoma; MEC, mucoepidermoid carcinoma; PGE2, prostaglandin E2.

## Abbreviations

COX	cyclooxygenase
HNC	head and neck cancers
OSCC	oral squamous cell carcinoma
HPV	human papillomavirus
OPSCC	oropharyngeal squamous cell carcinoma
PG	prostaglandin
NPC	nasopharyngeal carcinoma
HR	hazard ratio
EP	prostanoid receptors
PGE2	prostaglandin E2
VEGF	vascular endothelial growth factors
HNSCC	head and neck squamous cell carcinoma
AdCC	adenoid cystic carcinomas
MEC	mucoepidermoid carcinoma
TSCC	tongue squamous cell carcinoma
LSCC	laryngeal squamous cell carcinoma
OTSCC	oral tongue squamous cell carcinoma
HPSCC	hypopharyngeal squamous cell carcinoma
